# Effectiveness of Antiepidemic Measures Aimed to Reduce Carbapenemase-Producing Enterobacteriaceae in the Hospital Environment

**DOI:** 10.1155/2022/9299258

**Published:** 2022-04-26

**Authors:** Maria Pawlak, Katarzyna Lewtak, Aneta Nitsch-Osuch

**Affiliations:** Department of Social Medicine and Public Health, Medical University of Warsaw, Faculty of Medicine, Warsaw, Poland

## Abstract

**Purpose:**

The objective of this study was to evaluate the effectiveness of hospital-based antiepidemic measures aimed at limiting the spread of symptomatic infections and colonization with carbapenem-resistant *Enterobacteriaceae* (CPE), mainly NDM-producing *Klebsiella pneumoniae*, with particular emphasis on microbiological screening tests.

**Methods:**

This retrospective study was based on data from 168 hospitals under the supervision of the Provincial Sanitary and Epidemiological Station in Warsaw, Poland, in 2016–2017. Analysis of the effectiveness of antiepidemic procedures focused on the type of implemented antiepidemic procedures, the number of microbiological screening tests per year, the geographic location of the hospitals (inside or outside Warsaw), the timing of the screening tests (on admission to hospital or 48 hours later), and the results of the screening tests.

**Results:**

Rates of proper isolation of patients infected or colonized with an alarm pathogen including NDM-producing *K. pneumoniae* increased from 38.0% in 2016 to 49.5% in 2017 (*p* > 0.05). The number of screening tests performed increased by 88% from 68319 in 2016 to 128373 in 2017. The number of epidemic outbreaks of symptomatic infections caused by NDM-producing *K. pneumoniae* decreased from 11 in 2016 to 7 in 2017 in hospitals in Warsaw, where microbiological screening tests were performed. The number of outbreaks in hospitals outside Warsaw, where the screening tests were not performed or were limited, increased from 8 in 2016 to 24 in 2017.

**Conclusion:**

Screening tests increase the chance of detecting colonization by CPE. The implementation of microbiological screening decreased the risk of epidemic outbreaks of symptomatic infections caused by CPE.

## 1. Introduction

The high prevalence of multidrug-resistant microorganisms is a serious public health problem. In recent years, the emergence of carbapenemase-producing *Enterobacteriaceae* (CPE) has been a particular concern [1]. Different variants of carbapenemases have been reported, belonging to Ambler classes A, B, and D [2]. Risk factors for CPE infection are intensive care unit (ICU) stay, mechanical ventilation, indwelling devices, diabetes mellitus, use of multiple antimicrobial agents, administration of carbapenems, sepsis, and surgical intervention [3]. Infections caused by CPE are associated with mortality rates as high as 40–50% [4]. The European Survey of Carbapenemase-Producing *Enterobacteriaceae* estimated the prevalence of CPE (*Klebsiella pneumoniae* and *Escherichia coli*) at 1.3 per 10 000 hospital admissions [5]. In 2016, the National Reference Center for Antimicrobial Susceptibility in Poland reported the isolation of CPE from 3775 infected patients or carriers. Interestingly, 1461 (38.7%) of the isolates came from the Mazovian Voivodeship [6].

New Delhi metallo-*β*-lactamase (NDM) is a carbapenemase belonging to Ambler class B; it was identified in Sweden in 2008 and then spread rapidly worldwide [7]. *K. pneumoniae* is one of the most common producers of NDM. In the first quarter of 2017, the number of confirmed cases of NDM-producing *K. pneumoniae* in Poland increased by about 150% compared to the first quarter of 2016. During this period, the number of infections with NDM-producing *K. pneumoniae* in the Mazovian Voivodeship increased from 273 to 545 [8].

Due to the ability of CPE to spread easily and colonize patients in healthcare environments, prevention of the transmission of these microorganisms is crucial. The presence of CPE, including NDM-producing *K. pneumoniae*, in the hospital environment leads to increased treatment costs, prolonged hospitalization, therapeutic failures, and a higher risk of death [9]. Methods for reducing the transmission of pathogens include the following [10–12]:

- Rational antibiotic administration
- Isolation and cohorting of patients
- Hand hygiene
- Internal training and audits
- Microbiological diagnostics
- Minimizing use of invasive devices
- Screening

Microbiological screening to rapidly identify colonized or infected patients is essential to prevent the transmission of alarm pathogens, including NDM-producing *K. pneumoniae*, in the hospital setting. Microbiological screening tests are recommended on hospital admission and 48 hours after hospitalization. Screening on admission should be performed in all patients or in at-risk patients, such as those hospitalized within the last 12 months, those with a history of CPE colonization, those staying in long-term care facilities, and those who have had contact with medical care in countries with a high incidence of infections with NDM-producing *K. pneumoniae* (India, Pakistan, and North Africa) [12–14].

Due to the worsening epidemiological situation in the Mazovian Voivodeship in November 2015, a new definition for epidemic outbreaks caused by NDM-producing *K. pneumoniae* was proposed by the Provincial Sanitary and Epidemiological Station in Warsaw. According to the new definition, an outbreak caused by NDM-producing *K. pneumoniae* should be considered when at least two cases of CPE (the same species and the same carbapenemase) are found in the same hospital ward within 1 month. One of these cases may be colonization, whereas the previous definition required the occurrence of two symptomatic cases [13, 15].

The objective of this study was to evaluate the effectiveness of hospital-based antiepidemic measures aimed at limiting the spread of symptomatic infections and colonization with CPE, mainly NDM-producing *K. pneumoniae*, with particular emphasis on microbiological screening tests.

## 2. Materials and Methods

In this retrospective study, data were collected from hospitals located in Warsaw and the Mazovian Voivodeship in 2016–2017. We have included data from all wards of hospitals which sent voluntary reports to the Provincial Sanitary and Epidemiological Station in Warsaw on the elimination of epidemic outbreaks and a survey on the implementation of screening procedures, with particular emphasis on the time and number of microbiological screening tests. A report form is included in supplement 1. The reported data were extracted and analyzed using Excel software (Microsoft). Analysis of the effectiveness of antiepidemic procedures focused on the type of implemented antiepidemic procedures, the number of microbiological screening tests per year, geographic location (hospitals in Warsaw *vs*. hospitals outside Warsaw), timing of the screening tests (on the day of hospital admission *vs*. 48 hours after admission), and results of the screening tests.

## 3. Statistical Analysis

To assess the effectiveness of microbiological screening tests, differences in the prevalence of CPE-related outbreaks were compared between hospitals in Warsaw (where screening was implemented) and those outside Warsaw (where screening was limited or not implemented). The analysis included the number of microbiological screening tests per year. *p* values were calculated under an alternative hypothesis that assumed differences in the analyzed characteristics. The null hypothesis was rejected in favor of the alternative hypothesis for *p* values <0.05 (statistical significance *p* < 0.05). Odds ratios (ORs) and 95% confidence intervals (95% CIs) were calculated with Fisher's exact test. The nonparametric Chi-square (*χ*2) test was used for nominal variables. Statistical analyses of the results were performed using the statistical and analytical software STATISTICA 10.0 PL (Dell Inc. 2016) and SPSS Statistics (Statistical Package for the Social Sciences Statistics) version 26, IBM.

## 4. Results

The questionnaires were returned in 2016 from 96/168 (57%) hospitals of the Mazovian Voivodeship province, including 16/87 (19%) of Warsaw hospitals and 80/81 (98%) hospitals located outside of Warsaw. In 2017, the questionnaires were returned from 99/168 (59%) hospitals of Mazovian Voivodeship, including 19/87 (22%) of Warsaw hospitals and 80/81 (98%) hospitals located outside of Warsaw.

### 4.1. Isolation, Cohorting, Internal Audits, and Hand Hygiene

**Figure 1:**
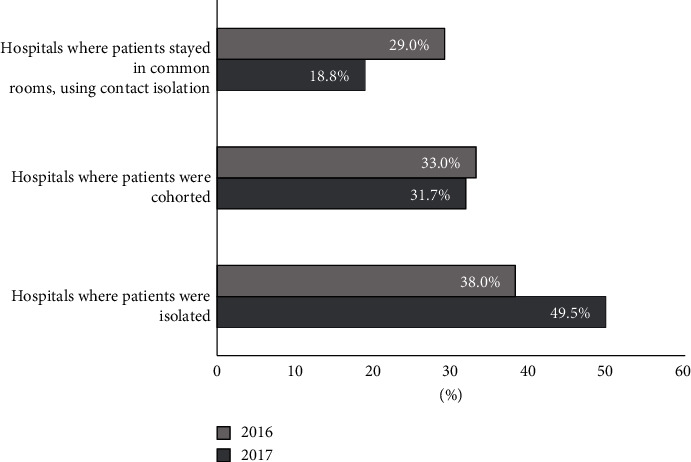
Use of patient isolation as an antiepidemic procedure in hospitals in the Mazovian Voivodeship in 2016–2017.

Proper isolation of patients infected or colonized with an alarm pathogen, including NDM-producing *K. pneumoniae*, takes place in less than 50% of hospitals. Although this percentage increased from 38.0% in 2016 to 49.5% in 2017, the difference between the years was not statistically significant (*p* > 0.05). If contact isolation was not possible, patients can be cohorted as long as they are colonized with the same bacterial species with an identical drug resistance mechanism. The percentages of hospitals cohorting patients and using contact isolation did not differ significantly between 2016 and 2017. Detailed data are presented in Figure 1.

The average number of hand disinfection procedures performed by medical personnel was 12.0 per patient per day in 2016, which increased slightly to 13.5 in 2017 (*p* > 0.05). The mean annual use of disinfectant per patient per day was similar between 2016 and 2017 (Table 1).

The number of internal audits performed in hospitals ranged from 1 to 9 per year, with an average of 4.8 in 2016 and 3.9 in 2017 (*p* > 0.05).

### 4.2. Microbiological Screening

Questionnaires about microbiological screening were completed by 96/168 (57%) hospitals in the Mazovian Voivodeship in 2016, including 16/87 (18%) hospitals in Warsaw and 80/81 (99%) hospitals outside Warsaw. In 2017, completed questionnaires were obtained from 99/168 (59%) hospitals in the Mazovian Voivodeship, including 19/87 (22%) hospitals in Warsaw and 80/81 (99%) hospitals outside Warsaw.

**Figure 2:**
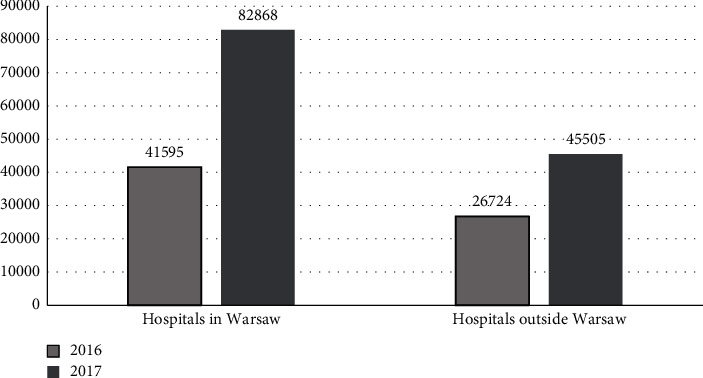
Number of microbiological screening tests performed in hospitals in the Mazovian Voivodeship in 2016–2017.

The number of performed screening tests increased by 88% from 68 319 in 2016 to 128 373 in 2017 (Table 2). The number of performed screening tests increased by 99% in hospitals in Warsaw and by 70% in hospitals outside Warsaw (Figure 2).

The increase in the number of screening tests performed resulted from an increase in both the number of tests performed on the day of admission to the hospital (by 71%) and those performed 48 hours after hospitalization (by 155%) (Table 2). In both analyzed years, most screening tests were performed on the day of admission: 60 812/68 319 (89%) of screening tests in 2016, and 109 248/128 373 (85%) in 2017 (Table 2). The difference in the percentage of screening tests performed on admission between 2016 and 2017 (89% *vs.* 85%) was not statistically significant (*p* > 0.05). Hospitals in Warsaw performed 678% more microbiological screening tests per hospital than hospitals outside Warsaw in 2016, and 667% more in 2017.

The increased number of screening tests was related to an increased number of positive results, ranging from a 128% increase on tests performed after 48 hours of hospitalization to a 145% increase on tests performed on admission to the hospital (Table 3). Positive results were obtained in 996 (1.5%) cultures in 2016 and in 2368 (1.8%) cultures in 2017 (Table 3). No statistically significant differences were found in the percentage of positive screening results between 2016 and 2017 (*p* > 0.05).

### 4.3. Epidemic Outbreaks

**Figure 3:**
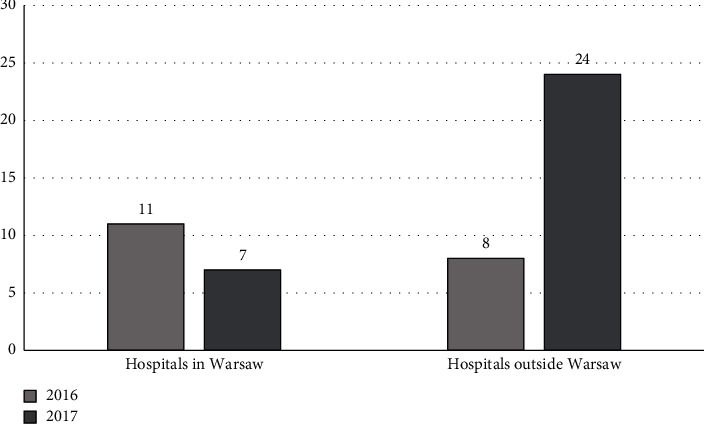
Number of epidemic outbreaks of symptomatic infections with NDM-producing *Klebsiella pneumoniae* in hospitals in the Mazovian Voivodeship in 2016–2017.

The number of epidemic outbreaks of symptomatic infections with NDM-producing *K. pneumoniae* decreased from 11 in 2016 to 7 in 2017 in hospitals in Warsaw, where microbiological screening tests were performed (Figure 3). The number of outbreaks in hospitals outside Warsaw, where screening tests were not performed or were limited, increased from 8 in 2016 to 24 in 2017 (Figure 3). The risk of epidemic outbreaks was significantly lower in hospitals in Warsaw, where the screening recommendations were implemented, than in hospitals outside Warsaw, where they were not (OR 0.63, 95% CI 0.21–0.87; *p* < 0.05).

## 5. Discussion

Hospital antiepidemic procedures aim to limit the spread of symptomatic infections and colonization caused by alarm pathogens. The extinction of an epidemic outbreak always requires the isolation of the infected or colonized patients, or the cohort of patients infected or colonized with the same pathogen. The results of our study showed that isolation of patients infected or colonized with CPE, including NDM-producing *K. pneumoniae*, took place in less than 50% of hospitals in the Mazovian Voivodeship in 2016–2017. This result should be a concern. For patients treated in the ICU, where it is difficult to provide a separate room, stationary isolation with a sanitary regime must be sufficient. Patients and carriers who require treatment in standard hospital wards should be strictly isolated, which is not usually possible due to hospital architecture [13].

Hand hygiene is important to prevent the transmission of hospital pathogens [16]. The results of the Point Prevalence Survey of Healthcare Associated Infections and Antimicrobial Use showed that use of alcohol-based hand disinfectants in Polish hospitals is lower than in European Union (EU) countries. Average use of hand disinfectant in Polish hospitals was 17.9 L per 1000 patient-days in 2014 and 16.9 L per 1000 patient-days in 2015 [17], compared with an average of 34.2 L per 1000 patient-days in participating EU countries and 32 L per 1000 patient-days in Germany [18]. These data indicate a lack of appropriate habits and noncompliance with recommendations in Polish hospitals. Results of our study indicated that the average number of hand disinfections has increased insignificantly over the analyzed years. In the study by Jaworski et al., the average number of hand disinfection procedures in the department of pediatric cardiac surgery was similar, but increased from 11.9 to 33 per person per day after staff training [19]. In other Polish observational studies, hand hygiene procedures were performed before patient contact in 5.2% of cases and after patient contact in 26.4% of cases [20, 21]. There is substantial evidence that enhanced adherence to hand hygiene reduces the risk of pathogen transmission [22].

According to Polish law, the responsibilities of the hospital infection control team include conducting internal audits and presenting the results and conclusions to the head of the hospital and the hospital infection committee [23]. The results of this study indicate that the number of internal audits ranged from 1 to 9 (on average, 4) per year in hospitals in the Mazovian Voivodeship in 2016–2017. This means that the frequency of internal inspections was too low in some hospitals, as the law requires two audits a year. Moreover, the average number of internal audits did not change significantly over the analyzed period. Performing internal audits is essential to reduce the transmission of pathogens responsible for hospital infections. Microbiological audits should include infection control policies in wards and departments, microbiological safety, cleanliness of the hospital environment, and an audit of standard healthcare equipment [24].

The spread of CPE in the hospital environment is an important public health problem. Procedures to reduce the risk of spreading CPE, including microbiological screening tests, are recommended by European societies [25, 26]. Microbiological screening tests are crucial in infection control programs, not only during epidemics and outbreaks but also as a routine prevention procedure [14]. Despite these recommendations, some hospitals in Poland do not perform microbiological screening tests. In 2016, 14% of healthcare entities in the Mazovian Voivodeship did not perform such tests at all, and 42% performed only a limited number (100–250 tests per quarter) [15]. Performing microbiological screening tests reduces the risk of nosocomial transmission of CPE. Our study clearly showed that the recommendations of the Sanitary Inspection were followed in hospitals in the Mazovian Voivodeship. The number of microbiological screening tests performed in the analyzed period increased by 88% (from 68319 in 2016 to 128373 in 2017). There was an increase in the number of microbiological screening tests performed both on the day of hospital admission (by 71%) and after 48 hours of hospitalization (by 155%). The performance of microbiological screening tests reduced the risk of infection outbreaks, as the risk of outbreaks was statistically significantly lower in hospitals in Warsaw, where screening recommendations were implemented, than in hospitals outside Warsaw, where they were not (*p* < 0.05).

The results of a study in New York, USA, showed a significant decrease in the transmission of NDM-producing *K. pneumoniae* after the implementation of an infection control program including microbiological screening in the ICU. The program included screening for gastrointestinal carriage of carbapenem-resistant *K. pneumoniae* and *Acinetobacter baumannii* and isolation of patients (in rooms at the far end of the ICU, separated only by curtains). The program also included intensive surface disinfection with isopropanol and a quaternary ammonium compound. The implemented rules reduced the average number of new cases of infections with carbapenemase-producing *K. pneumoniae* from 9.7 to 3.7 per 1000 patient-days per quarter [27]. In 2007, Israel implemented a national program aimed at limiting the spread of carbapenem-resistant *K. pneumoniae*. Planned activities include the identification and isolation of carriers. The monthly incidence of infections caused by carbapenem-resistant *K. pneumoniae* was 55.5 per 100 000 patient-days before the intervention and 11.7 cases per 100 000 patient-days after (*p* < 0.001) [28].

Enfield et al. described the efficacy of enhanced infection control measures at a 15-bed surgical trauma ICU. The measures included weekly education, disinfection, isolation, and cohorting of CPE carriers. After the intervention, the incidence of CPE in the surgical ICU was reduced from 7.77 to 1.22 cases per 1000 patient-days [29].

Hospital-based antiepidemic measures include hand hygiene, internal audits and education, patient isolation, cohorting, and microbiological screening. The results of our study and others indicate that enhanced infection control measures lead to a reduction in infections and outbreaks of CPE.

The main limitation of this study is that the analyses were based on data voluntarily submitted to the Provincial Sanitary Station in Warsaw, and not all hospitals submitted data. There is a need for healthcare facilities to be required to report data on microbiological screening tests for alarm pathogens, including CPE. This will allow to identify the scale of the problem and plan strategies to manage hospital-acquired infections. This approach can result in reduced infection rates and cost savings.

## 6. Conclusion

Implementation of standard infection control measures is effective in preventing the spread of CPE. Screening tests increase the chance of detecting colonization by CPE. The implementation of microbiological screening decreased the risk of epidemic outbreaks of symptomatic infections caused by CPE, including NDM-producing *K. pneumoniae*, which confirms the effectiveness of screening in reducing the risk of transmission in the hospital environment.

## Figures and Tables

**Table 1:** Hand disinfection as an antiepidemic procedure in hospitals in the Mazovian Voivodeship in 2016–2017.

Procedure	2016	2017	*p* value	95% CI
Mean number of hand disinfection procedures per patient per day	12.0	13.5	>0.05	0.71–16.28
Mean annual use of disinfectant per patient per day (mL)	40.5	40.2	>0.05	0.82–56.83

**Table 2:** Number of microbiological screening tests performed in hospitals in the Mazovian Voivodeship in 2016–2017.

	2016	2017	Change (%)
Number of screening tests	68 319	128 373	+88%
Number of screening tests on the day of admission to hospital	60 812	109 248	+71%
Number of screening tests performed after 48 hours of hospitalization	7507	19 125	+155%

**Table 3:** Number of positive results of microbiological screening tests performed in hospitals in the Mazovian Voivodeship in 2016–2017.

	2016	2017	Change (%)
Number of positive results	996/68319 (1.5%)	2369/128373 (1.8%)	+1373 (138%)

Number of positive results of screening tests performed on admission	577/60812 (0.9%)	1414/109248 (1.3%)	+837 (145%)

Number of positive results of screening tests performed after 48 hours of hospitalization	419/7507 (5.6%)	954/19125 (4.9%)	+535 (128%)

## Data Availability

The data that support the findings of this study are available on request from the corresponding author.

## Supplementary Materials

Supplementary Materials Supplementary material. Sample hospital questionnaire on implementation of screening procedures.
