# Supplementary material for: Effectiveness of Antiepidemic Measures Aimed to Reduce Carbapenemase-Producing Enterobacteriaceae in the Hospital Environment
Source: Can J Infect Dis Med Microbiol. 2022 Apr 26;2022:9299258. doi: 10.1155/2022/9299258 (PMC9072043; doi:10.1155/2022/9299258)
Supplement: Supplementary Materials — Supplementary material. Sample hospital questionnaire on implementation of screening procedures. [file 9299258.f1.docx]

Sample hospital questionnaire on implementation of screening procedures

Screening tests 2016/2017

| **Provincial Sanitary and Epidemiological Station** | **No.** | **Hospital** | **City** | **I quarter** | | | |
| --- | --- | --- | --- | --- | --- | --- | --- |
|  |  |  |  | **Number of screening tests performed on at-risk patients on hospital admission**  **(up to 48h)** | **Number of positive results** | **Number of tests performed on patients hospitalized over 48 h** | **Number of positive results** |
|  |  |  |  |  |  |  |  |
|  |  |  |  |  |  |  |  |
|  |  |  |  |  |  |  |  |
|  |  |  |  |  |  |  |  |

Screening tests 2016/2017

| **Provincial Sanitary and Epidemiological Station** | **No.** | **Hospital** | **City** | **II quarter** | | | |
| --- | --- | --- | --- | --- | --- | --- | --- |
|  |  |  |  | **Number of screening tests performed on at-risk patients on hospital admission**  **(up to 48h)** | **Number of positive results** | **Number of tests performed on patients hospitalized over 48 h.** | **Number of positive results** |
|  |  |  |  |  |  |  |  |
|  |  |  |  |  |  |  |  |
|  |  |  |  |  |  |  |  |
|  |  |  |  |  |  |  |  |

Screening tests 2016/2017

| **Provincial Sanitary and Epidemiological Station** | **No.** | **Hospital** | **City** | **III quarter** | | | |
| --- | --- | --- | --- | --- | --- | --- | --- |
|  |  |  |  | **Number of screening tests performed on at-risk patients on hospital admission**  **(up to 48h)** | **Number of positive results** | **Number of tests performed on patients hospitalized over 48 h.** | **Number of positive results** |
|  |  |  |  |  |  |  |  |
|  |  |  |  |  |  |  |  |
|  |  |  |  |  |  |  |  |
|  |  |  |  |  |  |  |  |

Screening tests 2016/2017

| **Provincial Sanitary and Epidemiological Station** | **No.** | **Hospital** | **City** | **IV quarter** | | | |
| --- | --- | --- | --- | --- | --- | --- | --- |
|  |  |  |  | **Number of screening tests performed on at-risk patients on hospital admission**  **(up to 48h)** | **Number of positive results** | **Number of tests performed on patients hospitalized over 48 h.** | **Number of positive results** |
|  |  |  |  |  |  |  |  |
|  |  |  |  |  |  |  |  |
|  |  |  |  |  |  |  |  |
|  |  |  |  |  |  |  |  |
